# Vitamin C Effect on Mitoxantrone-Induced Cytotoxicity in Human Breast Cancer Cell Lines

**DOI:** 10.1371/journal.pone.0115287

**Published:** 2014-12-22

**Authors:** Eliana Guerriero, Angela Sorice, Francesca Capone, Virginia Napolitano, Giovanni Colonna, Gabriella Storti, Giuseppe Castello, Susan Costantini

**Affiliations:** 1 Centro Ricerche Oncologiche di Mercogliano, Istituto Nazionale per lo Studio E la Cura dei Tumori “Fondazione Giovanni Pascale”, IRCCS, 80131 Naples, Italy; 2 Department of Biochemistry, Biophysics and General Pathology, Second University of Naples, 80138 - Naples, Italy; 3 Department of Onco-Hematology, S.G. Moscati Hospital, 83100 Avellino, Italy; National Cheng Kung University, Taiwan

## Abstract

In recent years the use of natural dietary antioxidants to minimize the cytotoxicity and the damage induced in normal tissues by antitumor agents is gaining consideration. In literature, it is reported that vitamin C exhibits some degree of antineoplastic activity whereas Mitoxantrone (MTZ) is a synthetic anti-cancer drug with significant clinical effectiveness in the treatment of human malignancies but with severe side effects. Therefore, we have investigated the effect of vitamin C alone or combined with MTZ on MDA-MB231 and MCF7 human breast cancer cell lines to analyze their dose-effect on the tumor cellular growth, cellular death, cell cycle and cell signaling. Our results have evidenced that there is a dose-dependence on the inhibition of the breast carcinoma cell lines, MCF7 and MDA-MB231, treated with vitamin C and MTZ. Moreover, their combination induces: i) a cytotoxic effect by apoptotic death, ii) a mild G2/M elongation and iii) H2AX and mild PI3K activation. Hence, the formulation of vitamin C with MTZ induces a higher cytotoxicity level on tumor cells compared to a disjointed treatment. We have also found that the vitamin C enhances the MTZ effect allowing the utilization of lower chemotherapic concentrations in comparison to the single treatments.

## Introduction

Breast cancer is the most common cause of cancer death among women (522.000 deaths in 2012) and the most frequently diagnosed cancer in 140 of 184 countries worldwide [1]. It is usually classified according to the expression of estrogen receptors (ER), progesterone receptors (PR), or human epidermal growth factor receptor (HER2) [2]. Most of the current successful therapies for breast cancer include anti-estrogen therapies, aromatase inhibitors, or Herceptin, by targeting these receptors [3]. Triple-negative breast cancers (TNBCs), which represent about 15% of cases, do not express any of these receptors, and, thus, are more difficult to treat with existing therapies as well as they are more likely to metastasize because of poorer prognosis [4], [5], [6].

Among the chemotherapic drugs often used for breast cancer treatment [7], there is Mitoxantrone (MTZ), a synthetic anti-cancer analog of anthracycline antibiotics. It has demonstrated significant clinical effectiveness in the treatment of human malignancies [8], and has been largely used in the treatment of tumors such as acute myeloid leukemia, non-Hodgkin's lymphoma, prostate, breast cancer as well as of the active forms of secondary progressive multiple sclerosis [9], [10]. The anti-cancer effect of MTZ is due to its ability to interact with DNA, where it forms a covalent complex with topoisomerase II that is able to prevent the rejoining of DNA strands during replication and, therefore, causing the breakage of DNA double-strand (DSB) [11], [12], [13]. As a consequence, MTZ inhibits DNA replication and RNA transcription, and, also, affects the cell cycle at various stages [14]. Despite its broad utilization, MTZ may induce cardiotoxicity, myelosuppression, leukopenia, renal insufficiency and extravasation irreversibly in a dose-dependent manner [15], [16]. For these reasons, it needs a careful evaluation to attenuate its side effects [13]. In recent years, the use of natural dietary antioxidants to minimize cytotoxicity and tissue damage by antitumor agents is gaining consideration [17]. Some natural substances, such as ascorbic acid (vitamin C or vit C), have been shown to improve the antineoplastic activity of some chemotherapeutic drugs [18]. Vit C is a water-soluble antioxidant and also an enzyme cofactor found in plants and some animals. It is a potent reducing agent that efficiently quenches potentially damaging free radicals produced through biological processes in many extracellular and intracellular reactions [19], [20], [21]. Several studies have demonstrated the beneficial employment of vit C in cancer treatment and a significant reduction in chemotherapy-induced adverse effects in patients receiving vit C [22]. Although larger studies will be needed to confirm a direct anticancer effect of vit C, its known ability to decrease chemotherapy-induced adverse effects should already make it a very valuable addition to chemotherapeutic regimens. In fact, a reduction in toxicity would allow patients to tolerate higher and potentially more effective doses of chemotherapy [23]. In this work, we have tested the vit C effect alone or combined with MTZ on two human breast cancer cell lines, TNBC (MDA-MB231) and MCF7, to analyze the dose-effect response of these combinations on tumor cell growth, as well on its death, its cycle and the signaling. Our results show that combined supplementations of vit C and MTZ have cytotoxic effect by apoptotic death, a mild G2/M elongation and activation of H2AX and mild PI3K pathways, and, hence, can be really useful for improving the chemotherapy treatments on breast cancer.

## Methods

### Cell cultures and treatments

Human breast carcinoma cell lines, MDA-MB231 and MCF7 (Lonza, Verviers, Belgium), were kept in culture and expanded at 37°C in a humidified atmosphere of 5% CO2 in culture medium DMEM (Dulbecco's Modified Eagle's Medium, Lonza) for MCF7 and RPMI 1640 (Lonza, B-4800 Verviers, Belgium) for MDA-MB231, supplemented with FBS (Invitrogen, Camarillo, CA, USA) at 10%, Penicillin/Streptomycin 100× (Euroclone, Devon, UK), Glutamax 100× (Invitrogen) and non-essential amino acids 100× (Invitrogen). Phosphate buffer (PBS phosphate buffered saline Ca^2+^ and Mg^2+^ free) and trypsin (Ca^2+^ and Mg^2+^ free) were supplied by Euroclone. The cells were plated 25×10^3^ for well in 96 well tissue culture plates and allowed to attach for 24h. Experiments were initiated when the cells reached 80% confluence. The cells were treated with MTZ (registered trademark ONKOTRONE; Baxter Healthcare S.A. 33 Vestey Drive, Mt Wellington, Auckland 1060, New Zealand) and vit C (galenic formulation, A.C.E.F.) for 48h. The drugs were dissolved in RPMI supplemented with 1% FBS at concentrations of 0.03, 0.07, 0.15, 0.30, 0.60, 1.20, 2.40 and 4.80 µM for MTZ and 0.03, 0.06, 0.12, 0.25, 0.50, 1, 2 and 4 mM for vit C for MDA-MB231 treatment. While the drugs were dissolved in DMEM supplemented with 1% FBS at concentrations of 0.07, 0.14, 0.29, 0.58, 1.17, 2.34, 4.68 and 9.36 and µM for MTZ and 0.09, 0.18, 0.37, 0.75, 1.50, 3, 6 and 12 mM for vit C during MCF7 treatment. The choice of these concentrations derived from Medline [24], [25]. Subsequently according to the results, combination treatment (co-treatment) with MTZ and vit C was carried out at IC50 (the median inhibitory concentration defined as the drug concentration at which cell growth was inhibited by 50%) doses obtained for 48h.

### Sulforhodamine B assay

After 48h of drugs exposition, cell survival/proliferation was measured in presence and absence of drugs in 96-well plates by a spectrophotometric dye incorporation assay using Sulforhodamine B (SRB). Cells were fixed with trichloroacetic acid (Sigma Aldrich, St. Louis, MO, USA) for 1h and after stained for 30 min with 0.4% (wt/vol) SRB (Sigma Aldrich) dissolved in 1% acetic acid. The number of viable cells was directly proportional to the protein bound-dye formation which was then solubilized with 10 mM Tris base solution pH 10.5 and measured by fluorometric assay ELISA at 540 nm (Bio-Rad, Hercules, CA, USA; Microplate Reader). All experiments were performed in duplicate and were repeated for three times [26]. The IC50 was assessed from the dose-response curves.

### Drug combination studies

Drug combination studies were based on concentration-effect curves generated as a plot of the fraction of unaffected (surviving) cells versus the drug concentration after 48 h of treatment. The values of triplicate experiments were analyzed and presented as means ± standard deviation (SD). Briefly, individually and combination of the two drugs equi-active doses (50∶50 cytotoxic ratio), were tested for 48 h. Synergism, additivity, and antagonism were quantified by determining the CI (combination index) calculated by the Chou-Talalay equation by means of the software CalcuSyn (Biosoft, Cambridge, UK) [27]. Assuming 0.9 as the cutoff, values of CI<0.9, 0.9<CI<1, or CI>1 indicate synergistic, additive, or antagonistic effect, respectively. The dose reduction index (DRI) represents the measure of how much the dose of each drug in a synergistic combination may be reduced at a given effect level, whereas compared with the dose of each drug, separately. The linear correlation coefficient (r) of the median-effect plot is considered a measure of the data conformity according to the mass-action law principle when an experimental measurement is assumed to be accurate. A r value equal to 1 indicates flawless conformity while a poor value may be the result of biological variability or experimental deviations. In all our experiments we calculated r values between 0.91 and 0.98, indicating a good compliance of the data.

### Apoptosis detection

The cells (1×10^6^) were harvested and washed twice with ice-cold PBS. Subsequently, the cells were labeled with “Annexin V & Dead Cell Assay kit” according to the manufacturer's instructions (Merck Millipore, Darmstadt, Germany). This assay is based on the phosphatidylserine (PS) detection on the apoptotic cells surface, using fluorescently labeled Annexin V in combination with the dead cell marker, 7-Aminoactinomycin D (7-AAD). We have calculated the apoptotic ratio by identifying four populations: i) viable cells, not undergoing detectable apoptosis: Annexin V (–) and dead cell marker (–), ii) early apoptotic cells: Annexin V (+) and dead cell marker (–), iii) late apoptotic cells: Annexin V (+) and dead cell marker (+), and iv) cells died through non-apoptotic pathway: Annexin V (–) and dead cell marker (+). The samples were counted by the Muse Cell Analyzer (Merck Millipore) and analyzed by a software provided by Merck Millipore.

### Cell cycle assay

The Muse Cell Cycle Assay uses a premixed reagent. This contains the nuclear DNA intercalating stain propidium iodide (PI) and RNAse A in a proprietary formulation. PI discriminates cells at different stages of the cell cycle, based on differential DNA content in the presence of RNAse to increase the specificity of DNA staining. The samples were centrifuged at 300xg for 5 min and after removing and discarding the supernatant, an appropriate volume of PBS was added to each tube (1 mL of PBS per 1×10^6^ cells). After centrifugation and removing of the supernatant, 1 mL of ice cold 70% ethanol was added to the re-suspending cell pellet in the residual PBS. The tubes were capped and frozen at −20°C for at least 3 h prior to staining. Ethanol-fixed cells were centrifuged at 300xg for 5 min at room temperature and the pellet was re-suspended in PBS. The cells were centrifuged again at 300xg for 5 min at room temperature, the supernatant was removed and discarded and cell pellet was re-suspended in 200 µL of Muse Cell Cycle Reagent and incubated for 30 min at room temperature, in the dark. Cell suspension samples were transferred to a 1,5 mL microcentrifuge tubes prior to analysis.

### Cell signaling pathways analysis

After 48 h of treatment, the cells (treated and untreated) were centrifuged at 300xg for 5 minutes and resuspended by adding 500 µl of 1X Assay Buffer and 500 µl of Fixation Buffer for one million cells (1∶1). The cells were incubated for 5 minutes on ice. After spinned down at 300xg for 5 minutes, the cells were permeabilized by adding 1 mL ice-cold Pemeabilization Buffer and incubated on ice for 5 minutes. The cells were centrifuged and resuspended in 450 µl 1X Assay Buffer. Then the cells were incubated with 10 µl of antibody (anti-H2AX and PI3K) for 30 minutes in the dark at room temperature. After that the cells were resuspended in 100 µl of 1X Assay Buffer and were centrifuged, they were resuspended in 200 µl of 1X Assay Buffer ad acquired on the Muse Cell Analyzer. The Muse H2AX Activation Dual Detection Kit includes two directly conjugated antibodies, a phospho-specific anti-phospho-Histone H2AX (Ser139)-Alexa Fluor 555 and an anti-Histone H2AX-PECy5 conjugated antibody to measure total levels of Histone H2AX. The Muse PI3K Activation Dual Detection Kit includes two directly conjugated antibodies, a phospho-specific anti-phospho-Akt (Ser473), Alexa Fluor®555 and an anti-Akt, PECy5 conjugated antibody to measure total levels of Akt. These two color kits are designed to measure the extent of H2AX phosphorylation relative to the total H2AX expression and of Akt phosphorylation relative to the total Akt expression in any given cell population. By doing such, the levels of both total and phosphorylated protein can be measured simultaneously in the same cell, resulting in a normalized and accurate measurement of H2AX and PI3K activation after stimulation.

### Statistical analysis

The differences of the effects on MDA-MB231 and MCF7 cells after three treatments with MTZ, vit C and their combination were compared by T-test. Values of p <0.05 were considered to be statistically significant. The statistical program Prism 4 (GraphPad Software, San Diego, CA, USA) was used.

## Results

### Cytotoxicity assay

**Figure 1:**
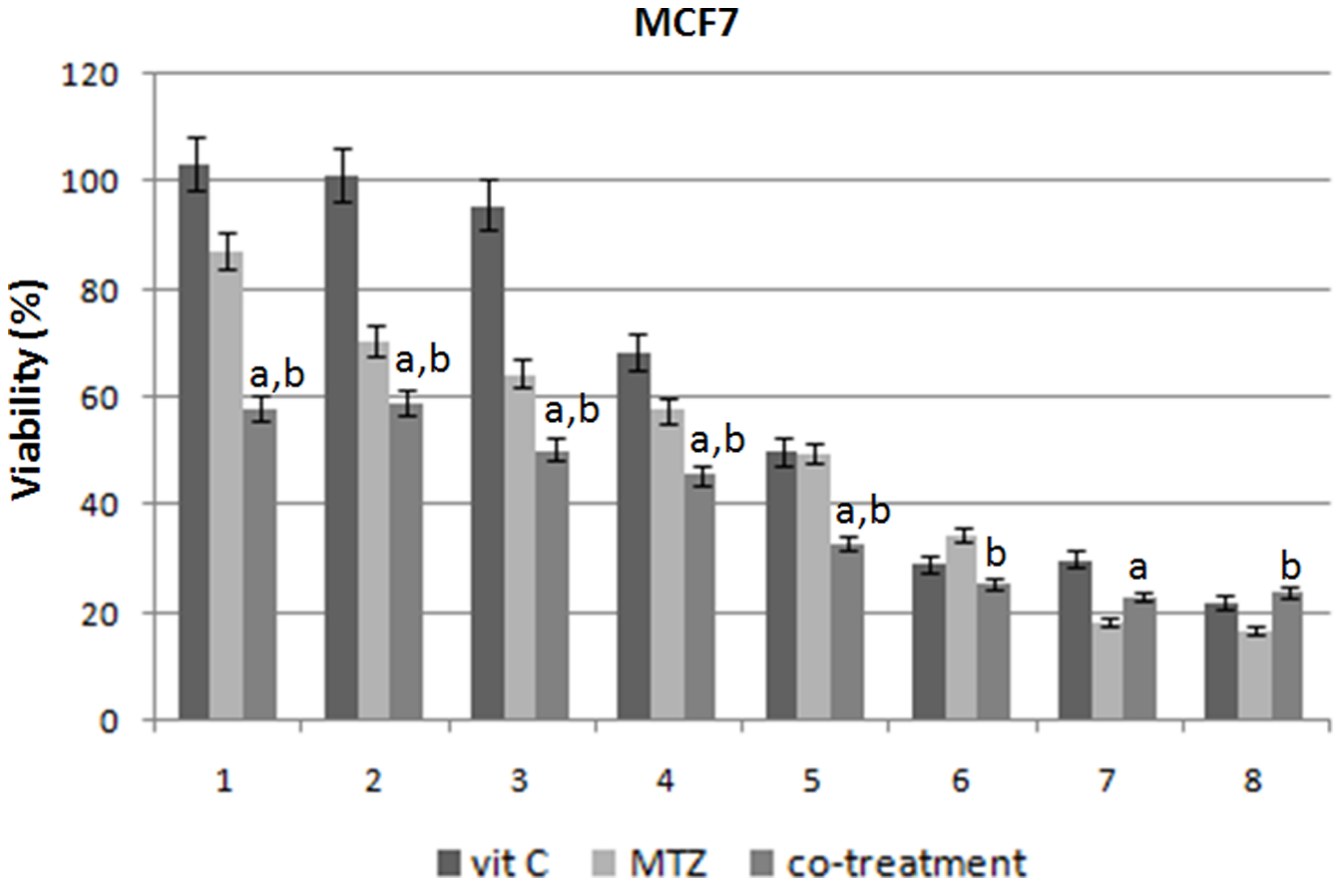
Cell viability %. The histograms show how changes the viability in MCF7 cell lines after MTZ, vit C, and their combination treatment for 48 h. Experiments were in triplicate. On the x-axis are showed the different drugs concentrations (DC): 1 (MTZ: 0.07 µM; vit C: 0.09 mM), 2 (MTZ: 0.15 µM; vit C: 0.19 mM), 3 (MTZ: 0.29 µM; vit C: 0.38 mM), 4 (MTZ: 0.58 µM; vit C: 0.75 mM), 5 (MTZ: 1.17 µM; vit C: 1.5 mM), 6 (MTZ: 2.34 µM; vit C: 3 mM), 7 (MTZ: 4.68; vit C: 6 mM), 8 (MTZ: 9.36; vit C: 12 mM) for MCF7 and 1(MTZ: 0.04 µM; vit C: 0.03 mM), 2 (MTZ: 0.75 µM; vit C: 0.06 mM), 3 (MTZ: 0.15 µM; vit C: 0.13 mM), 4 (MTZ: 0.3 µM; vit C: 0.25 mM), 5 (MTZ: 0,6 µM; vit C: 0.5 mM), and 6 (MTZ: 1,2 µM; vit C: 1 mM), 7 (MTZ:2,4; vit C: 2 mM), 8 (MTZ: 4,8; vit C: 4 mM) for MDA-MB231; on the y-axis: the viability percentage is presented as means ± standard deviation. Moreover, we indicate with “a” or “b” if the difference between cell viability after co-treatment and vit c or MTZ is statistically significant (with p-value<0.05).

**Figure 2:**
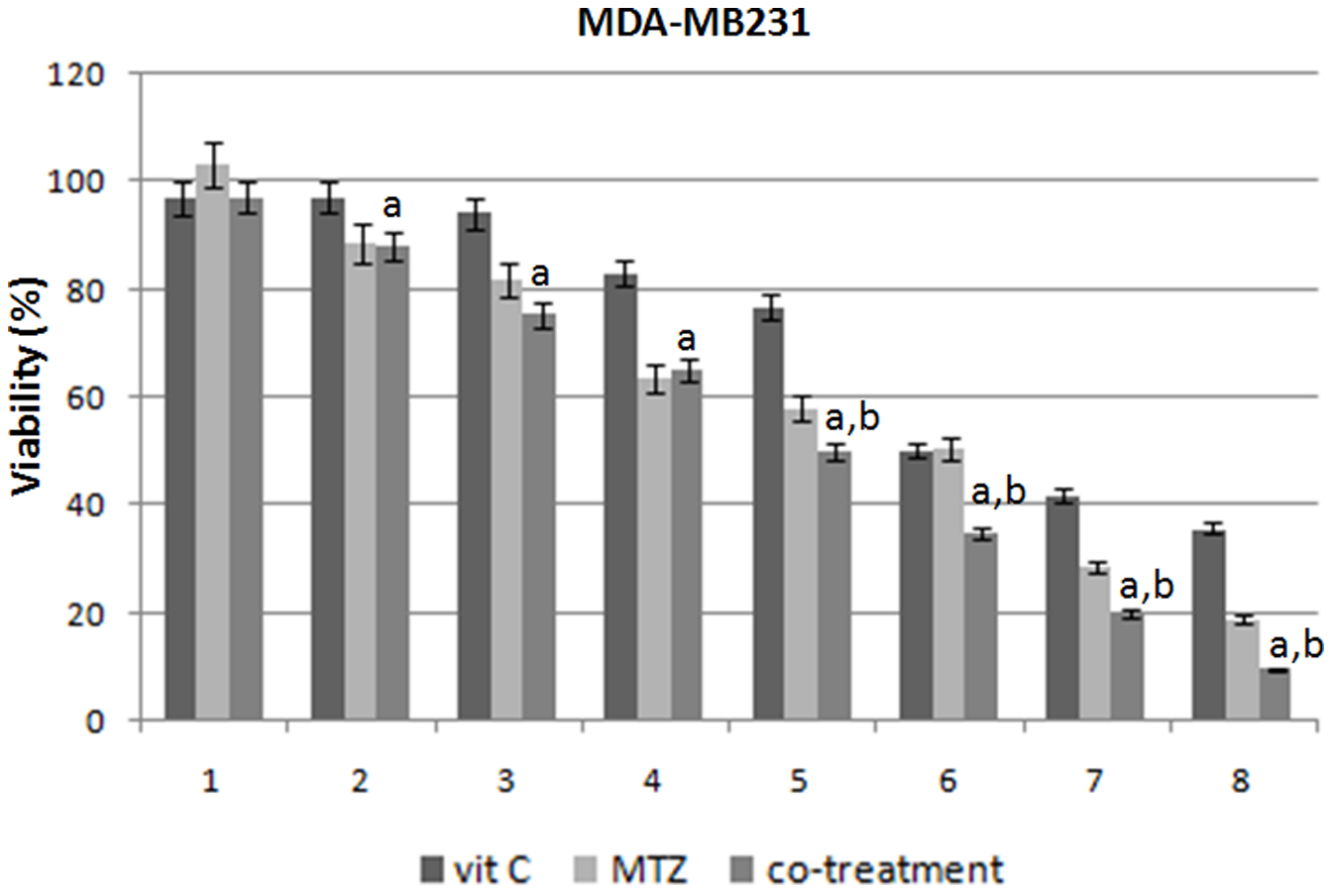
Cell viability %. The histograms show how changes the viability in MDA-MB231 cell lines after MTZ, vit C, and their combination treatment for 48 h. Experiments were in triplicate. On the x-axis are showed the different drugs concentrations (DC): 1 (MTZ: 0.07 µM; vit C: 0.09 mM), 2 (MTZ: 0.15 µM; vit C: 0.19 mM), 3 (MTZ: 0.29 µM; vit C: 0.38 mM), 4 (MTZ: 0.58 µM; vit C: 0.75 mM), 5 (MTZ: 1.17 µM; vit C: 1.5 mM), 6 (MTZ: 2.34 µM; vit C: 3 mM), 7 (MTZ: 4.68; vit C: 6 mM), 8 (MTZ: 9.36; vit C: 12 mM) for MCF7 and 1(MTZ: 0.04 µM; vit C: 0.03 mM), 2 (MTZ: 0.75 µM; vit C: 0.06 mM), 3 (MTZ: 0.15 µM; vit C: 0.13 mM), 4 (MTZ: 0.3 µM; vit C: 0.25 mM), 5 (MTZ: 0,6 µM; vit C: 0.5 mM), and 6 (MTZ: 1,2 µM; vit C: 1 mM), 7 (MTZ:2,4; vit C: 2 mM), 8 (MTZ: 4,8; vit C: 4 mM) for MDA-MB231; on the y-axis: the viability percentage is presented as means ± standard deviation. Moreover, we indicate with “a” or “b” if the difference between cell viability after co-treatment and vit c or MTZ is statistically significant (with p-value<0.05).

MTZ and vit C cytotoxic effects were evaluated on MCF7 and MDA-MB231 cell lines by SRB assay to identify the concentrations at which the cell growth was inhibited by 50%. After 48 h of treatment with MTZ and vit C, compared to non treated cells, MCF7 reached an inhibition corresponding to IC50 at 1.17 µM and 1.5 mM dose, whereas MDA-MB23 reached the same condition at 1.2 µM and 1 mM dose, respectively (Figs. 1 and 2). Taking advantage of the median drug effect analysis in calculating combination indexes (CIs), we have explored the anti-proliferative effects of MTZ and vit C combinations by testing equipotent doses of the two agents (50∶50 cytotoxic ratio). A synergistic effect with low CIs (CIs <0.9) was demonstrated when equipotent combination doses were used for both cell lines (Figs. 1 and 2, and Table 1). In details, after combined treatment we have achieved a dose reduction of 1.70-fold for MTZ and of 8.95-fold for vit C in MCF7 cells about IC50 values (DRI50) as well as of 2.04-fold and 3.16-fold for MTZ and vit C, respectively, in MDA-MB231 cells when compared with concentrations of the two drugs taken individually (Table 1).

**Table 1:** MTZ and vit C co-treatment induced a synergistic anti-proliferative effect compared to treatment with drugs administered individually as demonstrated by median drug effect analysis calculating the combination index (CI) and the dose redaction index (DRI) with CalcuSyn software.

Cell line	Treatment	CI_50_(±SD)	r (±SD)	DRI+ at IC_50_(±SD)	
				vit C	MTZ
MCF7	Vit. C + MTZ	0.70±0.05	0.97±0.02	8.95±0.04	1.70±0.09
MDA-MB231	Vit. C + MTZ	0.80±0.07	0.98±0.02	3.16±0.10	2.04±0.08

“r” is the linear correlation coefficient.

### Apoptosis Studies

Subsequently we investigated the ability of MTZ, vit C and their mixtures to induce apoptosis in MCF7 and MDA-MB231 cells. Treatments with only MTZ (1.17 µM and 1.2 µM in MCF7 and MDA-MB231 respectively) or vit C (1.5 mM and 1 mM in MCF7 and MDA-MB231 respectively) have clearly shown an apoptotic death around 81.5% and 67% in MCF7, and 70.3% and 43.8% in MDA-MB231, respectively. Simultaneous treatments with concentrations below the IC50 values of MTZ (0.29 µM in MCF7 cells; 0.60 µM in MDA-MB231 cells) and vit C (0.38 mM in MCF7 cells; 0.50 mM in MDA-MB231 cells) have shown a synergistic apoptotic effect. Although the values of apoptotic percentages of the co-treatment are comparable to those of the treatment with only vit C (68% and 67% in MCF7, and 49.2% and 43.8% in MDA-MB231, respectively), it is evident that the advantage, which is achieved with a combined formulation, is due to the fact that the doses of the chemotherapeutic agent are significantly reduced, and, precisely, from 1.17 µM to 0.29 µM in MCF7 and from 1.2 µM to 0.60 µM in MDA-MB231) (Table 2). However, it is important to underline that in MDA-MB231 the co-treatment has shown an increase in the percentage of necrotic cells (from 13.1% to 30.8%) when compared with the MTZ treatment (Table 2).

**Table 2:** Percentage of live, early apoptotic, late apoptotic, and dead cells expressed as mean ±standard deviation by the Muse Annexin V and Dead Cell assay in MCF7 and in MDA-MB231 cells.

MCF7	Live	Total apoptotic	Dead
**un-treated**	74.51±0.05	23.92±0.06	1.62±0.01
**MTZ**	17.23±0.02	81.53±0.08	1.33±0.01
**vit. C**	32.86±0.04	67.11±0.05	0.27±0.01
**co-treatment**	30.91±0.05	68.09±0.04	1.08±0.01

MDA-MB231			
**un-treated**	76.04±0.07	22.22±0.05	1.87±0.02
**MTZ**	16.62±0.01	70.34±0.07	13.11±0.05
**vit. C**	54.15±0.08	43.87±0.04	2.15±0.01
**co-treatment**	20.04±0.03	49.28±0.05	30.86±0.01

### Cell cycle

**Figure 3:**
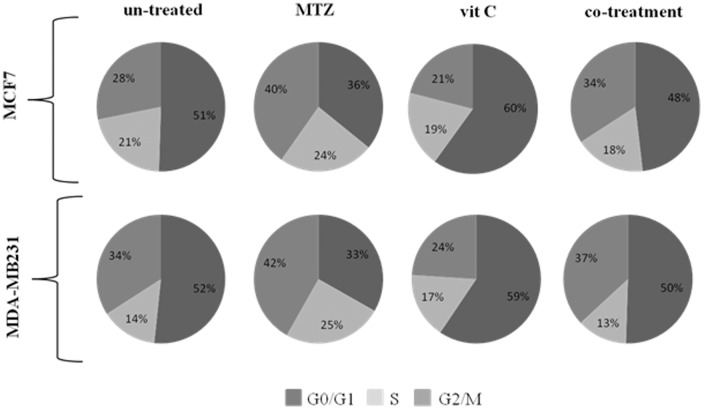
Cell percentages in G0/G1, S and G2/M phases in MCF7 and in MDA-MB231 cells.

We have evaluated the cell cycle of MCF7 and MDA-MB231 cells after single (1.17 µM and 1.2 µM MTZ dose and 1.5 mM and 1 mM vit C dose in MCF7 and MDA-MB231 respectively) and combined treatments (0.29 µM of MTZ plus 0,38 mM of vit C in MCF7; and 0,6 µM of MTZ plus 0.5 mM of vit C in MDA-MB231). In particular, the MTZ treatment has shown a G0/G1 phase slowdown and a G2/M elongation in both cell lines when compared with untreated cells (Fig. 3) while the vit C treatment has conversely shown a G0/G1 phase elongation and a G2/M slowdown (Fig. 3). Moreover, the co-treatment has induced a balancing effect on the cell cycle phases, showing only a mild G2/M elongation in both cell lines (Fig. 3).

### Cell signaling pathways analysis

We have also investigated the ability of MTZ, vit C, and of their combination, in modulating the activation pathways of H2AX and PI3K on both breast cancer cells. Cells were co-treated to concentrations below the IC50 values of MTZ (0.29 µM in MCF7 cells; 0.60 µM in MDA-MB231cells) and vit C (0.38 mM in MCF7 cells; 0.50 mM in MDA-MB231 cells). Then they were stained in multiplex either by anti-phospho-Histone H2AX (Ser139) or anti-Histone H2AX antibodies. Samples were counted by using the Muse Cell Analyzer and through the statistical values calculated for these assays, we determined the relative percentage of each population (inactive, active and not-expressing), compared to the total cell population. Regarding the H2AX signaling, we have obtained activations high and very similar in MDA, after stimulation with MTZ (86%) and vitamin C (91.4%) and after co-treatment (91.1%) whereas in MCF7 cells we have measured lower and different activations (35.7%, 60.8% and 42.1% for MTZ, vit C and co-treatment, respectively) (Table 3). On the other hand, the PI3K pathways showed in MDA-MB231 cells, no (0.90%), moderate (24.8%) and a weak (9.70%) activation, after treatment with MTZ, vit C and co-treatment, respectively, whereas in MCF7 we found a very weak activation in all three treatments (13.25%, 8.78% and 10.29%, respectively) (Table 3). Although the percentage of activation of the co-treatment compared to the treatment of MTZ and vitamin C alone, showed no significant difference (p-value >0.05), while the clear advantage of their association is given by the reduction in the dose of the chemotherapeutic drug (from 1.17 µM to 0.29 µM in MCF7 and from 1.2 µM to 0.60 µM in MDA-MB231), as we have already seen for apoptosis and cell cycle.

**Table 3:** H2AX and PI3K expression activation percentages for each cell population (inactive, active and not-expressing) compared to the total cell population.

	H2AX activation percentages			PI3K activation percentages		
MCF7	inactivated	activated	non-expressing	inactivated	activated	non-expressing
**un-treated**	92±2	6.8±1.8	1.1±0.8	96.8±2.8	1.7±0.8	1.5±0.9
**MTZ**	55.8±1.5	35.7±2.3*	8.5±1.1	70.9±1.5	13.2±1.5*	15.9±1.3
**vit. C**	36.6±2.2	60.8±1.8*	2.6±1.5	68.8±2.5	8.8±3.2*	22.4±2.5
**co-treatment**	49.3±1.5	42.1±1.5*	7.9±1.1	70.6±1.8	10.3±2.5*	19.1±1.8

MDA-MB231						
**un-treated**	73.2±1.6	8.5±2.1	17.9±1.1	97.9±2.6	0.4±0.02	1.7±0.5
**MTZ**	13.1±1.2	86±5*	0.9±0.05	95.1±2.5	0.9±0.5	4±0.5
**vit. C**	8.1±1.3	91.4±2.3*	0.3±0.02	68.9±3.3	24.8±3.8*	6.4±1.2
**co-treatment**	7.9±0.8	91.1±2.3*	0.9±0.05	73±2.5	9.7±2*	17.2±1.5

We report mean values ± standard deviation and indicate with * (p-value<0.05) if the activation difference in treated and untreated cells is statistically significant.

## Discussion

Recent studies have shown that pharmacological concentrations of vitamin C selectively kill tumor cells by induction of cytotoxicity, but not normal cells, which suggests its useful and beneficial use in the treatment of cancer [22], [28]–[30]. It was also previously reported that vit C induced cell cycle arrest and apoptosis in various tumor cells, such as lymphoma cells and leukemia cells [31], [32], melanoma cells [33], brain tumor cells [34], [35], prostate cancer cells [36], and stomach cancer cells [37]. On the other hand, MTZ is a synthetic anti-cancer analog of anthracycline that has demonstrated significant clinical effectiveness in the treatment of human malignancies [8], as well as of solid tumors [9], [10] by interacting with DNA, where it forms a covalent complex with topoisomerase II, which prevents rejoining of DNA strands during replication and induces DNA double-strand breaks (DSB) [11], [12], [13]. However, therapy with MTX shows significant side effects such as cardiotoxicity, myelosuppression, leukopenia, kidney failure and extravasation, with dose-dependent irreversibility. In this work we have studied the effect of vitamin C alone or in combination with MTZ on central biological functions (cell growth, death, cycle and signaling) of two cell lines of human breast cancer (MDA-MB231 and MCF7), in order to understand the dose-effect response of these molecules, alone or in combination, even considering that the MTZ is a component of the chemotherapy regimens indicated for the treatment of metastatic breast cancer [38]. Our data indicate that vit C, in synergy with MTZ, decreases the viability of cancer cells by inducing apoptosis in both cell lines of human breast cancer. However, pro-apoptotic effects of MTZ and vit C have previously been described [18], [25], [37]. In fact, it was reported that MTZ acts by intercalating DNA bases in breast cancer cells, thus causing cell death [25], while the vit C induces cell death through the apoptosis-inducing factor (AIF) in the human breast cancer cell lines (SK-BR3 and Hs578T) but not in a healthy breast cell line (Hs578) [37]; moreover, vit C exhibits cytotoxic activity at high concentrations in two human breast carcinoma cell lines (MCF7 and MDA-MB231) [18], effect that we have confirmed in this paper. Our results show that: i) vit C interferes with the cell cycle by inducing a G0/G1 phase elongation and a G2/M slowdown, ii) MTZ treatment shows a G0/G1 phase slowdown and a G2/M elongation in both cell lines, whereas iii) the co-treatment induces a mild G2/M elongation as visible after MTZ treatment alone.

However, several studies have already been performed on the effects of MTZ and vit C on the cell cycle. They have evidenced that MTZ alone induces cell cycle arrest and accumulation in late S and G2/M phase in two leukaemic cell lines (MOLT-4 and Jurkat) [11], whereas vit C induces a significant increase in the cell population at G1 cell cycle phase in B16F10 murine melanoma cell line [39]. No information is reported about their effect on cell cycle, alone and/or in combination, on breast cancer cell lines. Therefore, in our study we assessed for the first time that by means of a combination of MTZ and vit C, we can effectively induce a decreased cell viability of cancer cell lines through apoptosis and cell cycle modification, reducing both the dosage of the chemotherapeutic drug and of the vit C in comparison to the dosage of the two compounds, when used alone. However, although the values of apoptotic percentages of the co-treatment are comparable to those of the treatment with only vit C, it is evident that the advantage, which is achieved with a combined formulation, is due to the fact that the doses of the chemotherapeutic agent are significantly reduced, and, precisely, from 1.17 µM to 0.29 µM in MCF7 and from 1.2 µM to 0.60 µM in MDA-MB231) in which there is also an increase in the percentage of necrotic cells (from 13.1% to 30.8%) when compared with the MTZ treatment (Table 2).

Moreover, it is important to underline that in the literature it is reported that the vit C treatment induces a concentration dependent DSBs in SHIN3 human ovarian cancer cells, due to a time dependence of H2AX phosphorylation [40]–[41] which generates ROS, ATP depletion, and, thus, ATM/AMPK activation and mTOR inhibition, correlated to PI3K signaling pathway [23].

On the other hand, it is also reported that H2AX is phosphorylated by activation of ATM in response to DNA damage induced by MTZ in human lung carcinoma A549 cells, therefore, it plays a key role in DNA damage response, as well as it is additionally required either for the assembly of DNA repair proteins at those sites containing damaged chromatin that for activation of checkpoint proteins, which arrest the cell cycle progression [42]. In fact, the intensity of H2AX phosphorylation is correlated to the number of DSBs [43], [44], [45], because their production in cells that replicate DNA is a more effective inducer of apoptosis that DSB in G1 or G2M phase cells. However, some authors showed that the MTZ mediated inhibition of HIF-1a did not require Akt dephosphorylation, thus debating against the involvement of the PI3K/Akt/mTOR pathway [46].

Hence, we evaluated the effects of vit C and MTZ, alone or combined in a single formulation, on H2AX and PI3K signaling pathways. In agreement with the main mechanism of MTZ and vit C action and the inhibition of the nuclear enzyme topoisomerase II, we confirm the H2AX activation as well the mild PI3K activation, after both treatments. The different pathways of activation observed for MDA-MB231 and MCF7 human breast cancer cell lines to treatments are likely due to intrinsic difference of these two cell lines. Indeed, MCF7 cells are estrogen receptor alpha (ERalpha)-positive, weakly invasive and luminal epithelial-like, whereas MDA-MB231 cells are ERalpha-negative, highly invasive and fibroblast-like.

In overall, our studies have shown that: i) MTZ and vit C inhibit the cell growth of both breast carcinoma cell lines, MCF7 and MDA-MB231, in a dose-dependent manner, ii) their pooled effects require concentrations well below the correspondent IC**_50_**, and iii) the vit C is able to enhance the effects of the chemotherapic agent lowering its pharmacological concentration. However, to verify that we have identified the best concentrations for MTZ and vit C combination, we repeated the experiment using i) for vit C the concentrations used previously for MTZ (0.03, 0.07, 0.15, 0.30, 0.60, 1.20, 2.40 and 4.80 µM) and ii) for MTZ those used previously for vit C (0.03, 0.06, 0.12, 0.25, 0.50, 1, 2 and 4 mM). After having treated both MDA-MB231 and MCF7 cells with vit C µM concentrations being more low compared to those used previously, the cells resulted completely live (S1 Fig.) related to cell viability of MCF7 and MDA-MB231 cell lines. On the other hand, after having treated both MDA-MB231 and MCF7 cells with MTZ mM concentrations being more high compared to those used previously, the cells are enough completely died and presented a very low viability percentage (S1 Fig.). On the basis of these results we can conclude that the concentrations used in our experiment are those that permit to obtain the best results.

Hence, we propose the combined use of the MTZ and vit C as pharmacological formulation able to reduce from two to four times the IC**_50_** of the antineoplastic drug alone. We think that this should be a relevant improvement in the breast cancer treatment due to the well-known general toxicity of the chemotherapy agents, which often limit their dose and, at times, this is precisely the reason for chemotherapy interruption. However, further in vivo tests of this combination will be necessary to check whether it may be a better tool in the clinical practice for the treatment of breast cancer.

## Supporting Information

S1 Fig Cell viability (%) for MCF7 (A) and MDA-MB231(B) cell lines after vit C (A) and MTZ (B) treatment for 48 h. Experiments were in triplicate.
(DOC)
