# Supplementary material for: Vitamin C Effect on Mitoxantrone-Induced Cytotoxicity in Human Breast Cancer Cell Lines
Source: PLoS One. 2014 Dec 22;9(12):e115287. doi: 10.1371/journal.pone.0115287 (PMC4274052; doi:10.1371/journal.pone.0115287)
Supplement: S1 Fig — Cell viability (%) for MCF7 (A) and MDA-MB231(B) cell lines after vit C (A) and MTZ (B) treatment for 48 h. Experiments were in triplicate. (DOC) [file pone.0115287.s001.doc]

**A**


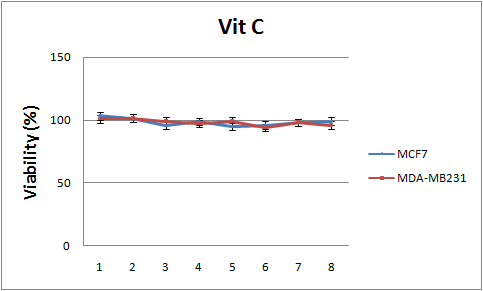


**B**


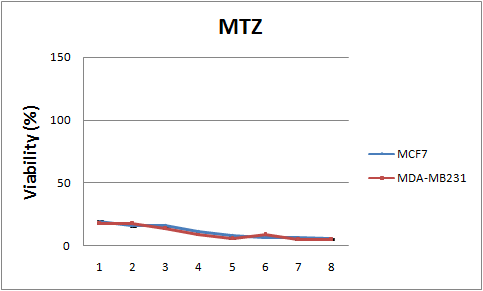


**Figure S1.** Cell viability (%) for MCF7 (A) and MDA-MB231(B) cell lines after vit C (A) and MTZ (B) treatment for 48 h. Experiments were in triplicate.
